# Prognosis Research Strategy (PROGRESS) 3: Prognostic Model Research

**DOI:** 10.1371/journal.pmed.1001381

**Published:** 2013-02-05

**Authors:** Ewout W. Steyerberg, Karel G. M. Moons, Danielle A. van der Windt, Jill A. Hayden, Pablo Perel, Sara Schroter, Richard D. Riley, Harry Hemingway, Douglas G. Altman, for the PROGRESS Group

**Affiliations:** 1 Department of Public Health, Erasmus MC, Rotterdam, Netherlands; 2 Julius Center for Health Sciences and Primary Care, UMC Utrecht, Utrecht, Netherlands; 3 Arthritis Research UK Primary Care Centre, Keele University, Keele, United Kingdom; 4 Department of Community Health and Epidemiology, Dalhousie University, Halifax, Nova Scotia, Canada; 5 London School of Hygiene & Tropical Medicine, London, United Kingdom; 6 BMJ, BMA House, Tavistock Square, London, United Kingdom; 7 School of Health and Population Sciences, University of Birmingham, Birmingham, United Kingdom; 8 Department of Epidemiology and Public Health, University College London, London, United Kingdom; 9 Centre for Statistics in Medicine, University of Oxford, Oxford, United Kingdom

## Abstract

In this article, the third in the PROGRESS series on prognostic factor research, Sara Schroter and colleagues review how prognostic models are developed and validated, and then address how prognostic models are assessed for their impact on practice and patient outcomes, illustrating these ideas with examples.

**Summary Points**

- The PROGRESS series (http://www.progress-partnership.org) sets out a framework of four interlinked prognosis research themes and provides examples from several disease fields to show why evidence from prognosis research is crucial to inform all points in the translation of biomedical and health related research into better patient outcomes. Recommendations are made in each of the four papers to improve current research standards.
- What is prognosis research? Prognosis research seeks to understand and improve future outcomes in people with a given disease or health condition. However, there is increasing evidence that prognosis research standards need to be improved.
- Why is prognosis research important? More people now live with disease and conditions that impair health than at any other time in history; prognosis research provides crucial evidence for translating findings from the laboratory to humans, and from clinical research to clinical practice.
- Prognostic models use multiple prognostic factors in combination to predict the risk of future clinical outcomes in individual patients. A useful model provides accurate predictions that inform patients and their care givers, supports clinical research, and allows for informed decisions to improve patient outcomes.
- Prognostic model research has three main phases: model development (including internal validation), external validation, and investigations of impact in clinical practice. Although many prognostic models are proposed, relatively few are currently used in clinical practice.
- Most publications on prognostic models describe model development, a small number report external validation studies, and only very few consider clinical impact or usefulness.
- Reliable models for clinical practice are more likely to be obtained when they are:
  1. Developed using a large, high quality dataset
  2. Based on a study protocol with a sound statistical analysis plan
  3. Validated in independent datasets obtained from different locations
- When accurate prognostic models are identified, impact studies are required to investigate their influence on decision making, patient outcomes, and costs.
- The performance of prognostic models may wane over time, possibly as diagnosis or treatments change. Rather than always developing new models from scratch, researchers should consider whether existing models can be improved by recalibration or adding novel predictors such as new biomarkers or results from new imaging techniques.
- The other papers in the series are:
  1. PROGRESS 1: *BMJ* 2013, doi:10.1136/bmj.e5595
  2. PROGRESS 2: *PLOS Med* 2013, doi:10.1371/journal.pmed.1001380
  3. PROGRESS 4: *BMJ* 2013, doi:10.1136/bmj.e5793

*Prognostic models are abundant in the medical literature yet their use in practice seems limited. In this article, the third in the PROGRESS series, the authors review how such models are developed and validated, and then address how prognostic models are assessed for their impact on practice and patient outcomes, illustrating these ideas with examples.*

The first two papers in this series focus on the variability in prognostic endpoints given specific startpoints [1] and on the search for factors that are associated with these endpoints [2]. Adequate prediction of prognostic endpoints, however, generally requires multiple prognostic factors (variables, predictors, or markers).

A prognostic model is a formal combination of multiple predictors from which risks of a specific endpoint can be calculated for individual patients. Other names for a prognostic model include prognostic (or prediction) index or rule, risk (or clinical) prediction model, and predictive model. For an individual with a given state of health (startpoint), a prognostic model converts the combination of predictor values to an estimate of the risk of experiencing a specific endpoint within a specific period. Ideally this produces an estimate of the absolute risk (absolute probability) of experiencing the endpoint, but it may instead provide a relative risk or risk score [3]–[5]. A well known, simple example is the Nottingham Prognostic Index (see Box 1) [6], which gives a score that relates to the survival probability of a woman with newly diagnosed breast cancer based on a combination of tumour grade, number of involved lymph nodes, and tumour size. Survival curves can be plotted for risk groups derived from the model, analogous to those for different values of a single prognostic factor shown in paper 2 of this series [2]. Figure 1 shows such curves for four risk groups derived from a prognostic model for renal outcome in IgA nephropathy. Such separation into risk groups is visually pleasing but disguises the large variation across groups in the actual event times of individuals. Using prognostic models to make predictions for individual patients is more accurate and so is often preferred to risk grouping, although risk groups may inform treatment choices and enable stratification for risk severity in clinical trials. Some prognostic models are accessible as web tools. For example, Figure 2 shows the predicted probability of death within 14 days and of death or severe disability at six months for a specific patient admitted to hospital with traumatic brain injury [7].

**Box 1. The Nottingham Prognostic Index (NPI) [6]**

The NPI combines tumour size, whether the cancer has spread to the lymph nodes, and the grade of the cancer to produce a risk score for women with newly diagnosed breast cancer. The formula is
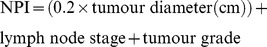
where:

Lymph node stage is scored as 1 (no nodes affected), 2 (≤3 glands affected), or 3 (>3 glands affected).

Tumour grade is scored as 1, 2, or 3.

A lower score suggests a good outcome.

**Figure 1:**
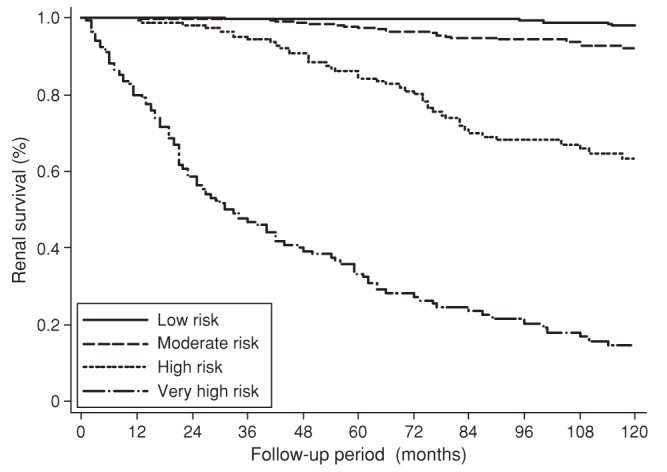
Kaplan-Meier survival curves for four risk groups derived from a prognostic model that provides a score to predict renal outcome in IgA nephropathy (reproduced from Goto et al [83]).

**Figure 2:**
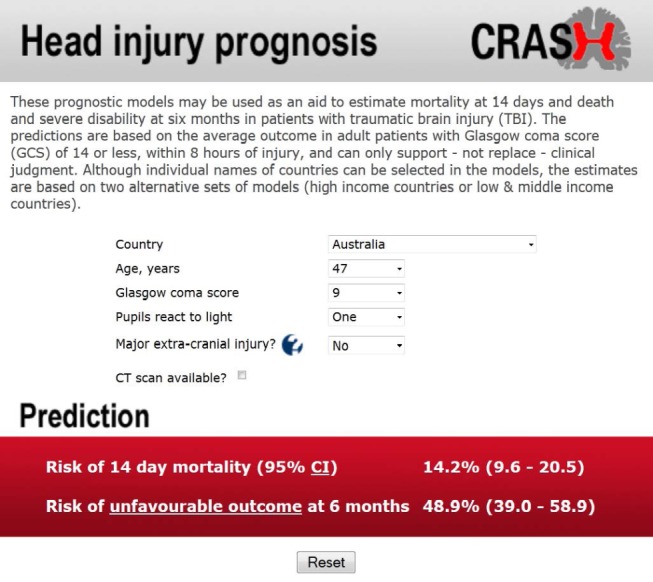
Web tool for prognosis of patients with head injury (CRASH trial) (reproduced from Perel et al [7] with permission).

## Aims of This Paper

In a previous *BMJ* series [4],[8]–[10] we described methods of developing a prognostic model, its external validation in a new setting, and evaluation of its clinical impact. We here discuss potential bottlenecks in these stages of evaluation to ensure that good prognostic models become clinically useful. We provide illustrative examples from oncology, cardiovascular disease, musculoskeletal disorders, and trauma (Table 1). We also consider the desirability of improving an existing model by incorporating novel prognostic factors or (bio)markers [11]–[13].

**Table 1:** Examples of the development, validation, and impact of prognostic models.

Name of prognostic model	Development	Validation	Impact
Nottingham Prognostic Index	Survival in 387 women with primary, operable breast cancer [6],[74]	Many studies, including an external validation in 9149 Danish patients [75]	Cited in guidelines. Survey indicated use in many centres to decide on adjuvant chemotherapy [76]. Modelling study for cost effectiveness analysis [76].
Örebro Musculoskeletal Pain Screening Questionnaire	Acute and subacute back pain in 142 workers [20]	At least 11 studies (median study size 123, range 45–298) [77]–[79]	Cited in guidelines and websites [80],[81]. Used to select trial participants [82].
CRASH/IMPACT	6 month outcome after traumatic brain injury (n = 10 008 for CRASH, n = 8530 for IMPACT) [43]	Cross-validation of CRASH on IMPACT and vice versa [43]	Cited as source of prognostic risk estimation [7]. Used to select trial participants and in analysis of randomised controlled trials.
Manchester Triage System	Urgency classification system by experts [21]	16 735 children in 2 Dutch hospitals [58]	Widely cited in most Western guidelines. Widely implemented, even before publication.

**Figure 3:**
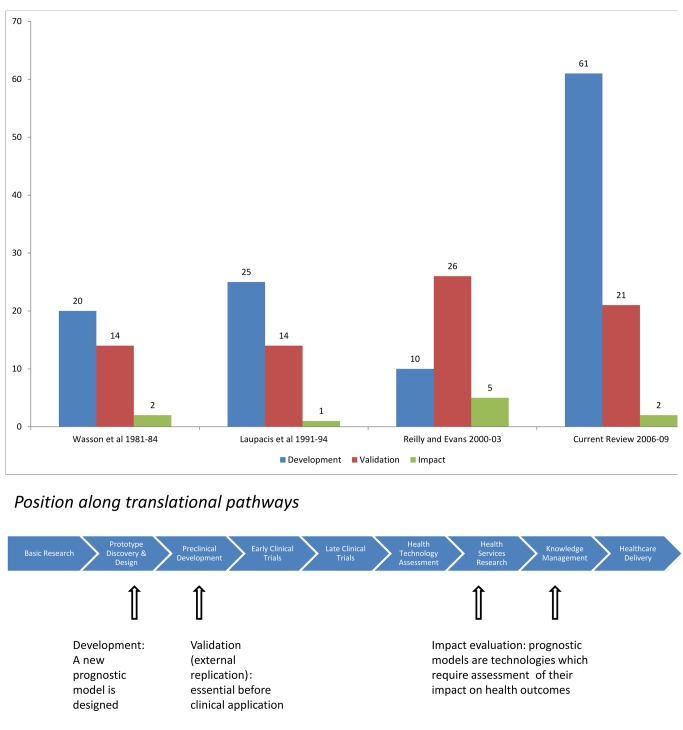
Distribution of published articles describing model development, validation, and impact assessment in four reviews (see Text S1). Path element adapted from Chart 7.1 in the Cooksey report (2006) http://bit.ly/Ro27rL (made available for use and re-use through the Open Government License).

We present findings of a systematic review of six leading general medical journals to obtain information about the number and nature of publications in 2006–09 reporting the development, validation, or assessment of impact of a prognostic model (see Text S1). Prognostic models are abundant in the medical literature [14]–[17], but few of the models are implemented or used in clinical practice [18]. Worse still, few models are evaluated for their impact on health outcomes, as shown in Figure 3.

## Why Are Prognostic Models Important?

Prognostic models are important at different stages in pathways leading to improvements in health (Figure 3, lower panel). The use of prognostic models ties in with the strong movement towards stratified medicine, where decisions regarding treatment choices are informed by an individual's profile of prognostic factors. Prognostic models aim to assist (not replace) clinicians with their prediction of a patient's future outcome and to enhance informed decision making with the patient.

The results from randomised therapeutic trials can be used to estimate how a specific treatment would modify a patient's estimated prognosis. Under the common assumption that a particular treatment has a constant relative benefit across all risk groups, the absolute treatment benefit depends on a person's predicted risk of the outcome without treatment [19]. Expensive therapies or those with harmful potential side effects may thus be reserved for those at higher risk, as estimated by a prognostic model.

Some prognostic models are used in clinical practice without being identified as such, such as the Apgar score for assessing the wellbeing of newborn babies [24]. Other examples of well used prognostic models include the Nottingham Prognostic Index [6], the Örebro Musculoskeletal Pain Screening Questionnaire to help clinicians identify patients with low back pain at risk of poor recovery [20], and the Manchester Triage System to assign priority based on clinical need among patients visiting an emergency department [21] (see Table 1). A prognostic model can thus be seen as an intervention that requires preclinical development, validation, and subsequent evaluation of its impact on health outcomes and cost effectiveness of care.

Prognostic models are also important to help improve the design and analysis of randomised therapeutic trials [22],[23], and to adjust for case mix variation in health services research [24], such as in understanding variations in patients' outcome across hospitals [25].

## Key Steps to Making Prognostic Models Useful

### Developing a Good Prognostic Model

The decision to develop a new prognostic model will be stimulated by a specific clinical uncertainty. Essential components are the startpoint and the specific outcome (endpoint). Methods for developing models have been widely discussed [3],[5],[10] and will not be described in detail here. In brief, prognostic models are usually and perhaps ideally derived with multivariable regression techniques on data from (prospective) cohort studies. Logistic and Cox regression modelling are most often used for short term and long term dichotomous outcomes (event occurrence yes/no) respectively. Important issues to be addressed include dealing with missing data [26], modelling continuous prognostic factors [27],[28], the complexity of the model [29], and checking the model assumptions. An important aim is to avoid statistical overfitting, especially when selecting from many candidate prognostic factors in a relatively small dataset.

Before any prognostic model might be adopted in practice it is necessary to show that it provides predictions that are valid outside the specific context of the sample that was used for model development (external validation) and ideally has real clinical impact. Of 86 papers published in leading general medical journals in 2006–09 that reported studies of prognostic models, the vast majority (n = 61) described the development of a prognostic model. The dearth of external validity and impact studies indicates no sign of improvement over earlier, similar reviews (see Figure 3 and Text S1).

### Validating a Prognostic Model

The predictive performance of a model estimated on the development data is often optimistic, related to multiple testing with a limited sample size [3],[5],[30],[31]. A newly developed prognostic model needs to be validated with patient data not used in the development process and preferably selected from different settings (external validation) [8],[32]–[34]. Indeed, the performance in such a validation study is arguably all that matters, and how a model was derived is of little importance if it performs well. Validation studies provide estimates of a model's ability to discriminate between patients with different outcomes and of the agreement between predicted and observed risks [35].

Our review found 21 publications which described external validation of a prognostic model (Figure 3 and Text S1). Among these, 18 included a geographical external validation (that is, validity of predictions for patients in another geographical area) and three a temporal external validation (validity in the same location at a later time) [32]. Validation of a prognostic model in a random part of the development cohort was common (14 of the 61 development studies). However, this approach (commonly referred to as internal validation) is statistically inefficient and methodologically weak since no difference in time or place exists other than by chance [8],[36].

For the Nottingham Prognostic Index, predictive performance has been tested in many external, often large, validation studies [37]. The Örebro Musculoskeletal Pain Screening Questionnaire [20] has been validated in several independent patient samples by multiple research groups, with 11 external validation studies up to 2009 (Table 1).

### Evaluating the Impact of a Prognostic Model on Clinical Practice and Outcomes

A prognostic model can influence patient outcome or the cost effectiveness of care only when changes in clinical management are made based on the prognostic information provided [9],[33]. Prognostic models have a cost in their implementation and might even have adverse consequences on clinical outcomes if they lead to decisions that withhold beneficial treatments (such as from people deemed by the model to be at low risk). Convincing evidence for the impact, positive or negative, of using prognostic models on patient outcome is hard to come by [9]. Our systematic review identified only two published analyses of the impact of prognostic models (Figure 3 and Text S1). A failure to recognise prognostic models as health technologies may be one reason why impact studies are lacking.

Most prognostic models are developed and validated with data from a single cohort of patients. Assessment of the impact of a model on decision making and patient outcome requires a comparative study [9],[33]. Here two groups (cohorts) need to be compared, one in which usual care is provided without the use of the model and another group in which model predictions are made available to doctors and other health professionals to guide treatment decisions. This comparison is scientifically strongest in a (cluster) randomised trial. An example is the STarTBack trial, in which primary care patients with back pain were randomised to receive either stratified care based on their risk of future disability or non-stratified best care. The results showed a significantly larger reduction in disability as well as cost savings in the group receiving stratified care compared with the control group [38].

Randomised trials are expensive and time consuming, and other approaches are possible. One can compare clinicians' decision making and patient outcomes observed in a time period before a model was introduced versus a time after it became available. An example of such a before and after study is an investigation of the effect of using the Nottingham Prognostic Index on the decision to treat women with adjuvant chemotherapy, resulting in modest effects on survival after its implementation [39]. However, potential time effects such as changes in current treatments should always be considered [9]. It is therefore desirable to include control practices that continue to deliver usual care in the time after implementation.

Alternative designs are necessary when there is a long time lag between the moment of prognostication (use of the model) and patient outcome or when outcomes are relatively rare. First, if a model has been well developed and validated, decision analytical modelling can be used to combine information on model predictions with information about the effectiveness of treatments from randomised trials or meta-analyses. If such modelling does not indicate improved outcome or favourable cost effectiveness, a long term randomised impact study may not (yet) be warranted. An example is a modelling study on the cost effectiveness of using various risk scores (with and without novel biomarkers) in patients with stable angina [40]. This study found that prioritising coronary surgery according to a prognostic model based on simple, readily available biomarkers was likely to be cost effective.

Another option is a cross sectional study with physicians' decisions as primary outcome [9],[41]. Clinicians or patients are randomised to either have or not have access to predictions from the prognostic model, and their therapeutic or other management decisions are compared. In another design, clinicians can be asked to decide on treatment or patient management before and after being provided with a model's predicted probabilities. This design has been used to assess the effect of using an additional test on medical decision making, such as 18-fluoro-deoxyglucose positron emission tomography (FDG-PET) to guide decisions on brain surgery [42].

For traumatic brain injury, no study has evaluated clinical impact, although many claim that the predictions from the models can be used to inform patients and relatives regarding prognosis. The CRASH and IMPACT models [7],[43] were based on large numbers of patients (n = 10 008 for CRASH, n = 8535 for IMPACT) and were well validated, but their application lies predominantly in research [44], in particular the design and analysis of randomised trials [23]. Impact on decision making for individual patients is less likely since predictions are not sufficiently certain to guide treatment limiting decisions [45].

The use and potential impact of prognostic models may be reflected in citations in practice guidelines and websites. That is the case for the Nottingham Prognostic Index, which is widely cited and included in the National Institute for Health and Clinical Excellence (NICE) guidelines of 2009 (Table 1). The evidence for its impact is still scarce, however. The use of Örebro Musculoskeletal Pain Screening Questionnaire is recommended in several clinical practice guidelines and on several websites (such as Work Cover Australia (http://www.workcover.nsw.gov.au) and the Australia Transport Accident Commission (http://www.tac.vic.gov.au)). Again, empirical evidence of its impact on physicians' decision making, let alone patient outcomes, is lacking.

### Updating a Prognostic Model

Updating a model is often desirable [5],[9],[46]–[48]. In particular, some systematic miscalibration is common for predictions obtained from prognostic models in settings that differ from that of the development sample. Updating methods include recalibrating the model to the new setting or investigating the addition of new prognostic factors, including biomarkers, to an existing model [46]. Ideally there should be an ongoing process of model validation and updating [5],[9],[46]–[48].

The contribution of genomic, proteomic, or metabolomic measures and new imaging tests over and above established prognostic factors is a key issue in current prognostic research [41],[49]. For example, a simple model for patients with traumatic brain injury that included just three strong prognostic factors was extended with computed tomography results in a second stage, and laboratory test results in a third stage [43]. The more extended models yielded more refined predictions and better discrimination. Various novel markers have been considered for their potential to improve the Nottingham Prognostic Index (Table 1).

The importance of assessing the impact of new markers on the accuracy of a model is widely agreed, but how best to quantify any changes in prediction is an active topic of methodological research [11]–[13]. The recent trend when comparing models is to consider the extent of reclassification of individual patients between risk groups rather than using global measures of discrimination such as the area under a receiver operating characteristic (ROC) curve [12],[50]. These different statistics are mathematically related, however [51],[52].

The addition of new markers may yield only marginal benefit [53]. Because standard models generally include important predictors, the independent effects of new prognostic factors need to be quite strong before a clinically useful improvement is achieved [54]. For example, adding two markers to a prediction model for patients with heart failure led to 342 (15%) of the 2345 patients initially classified as having a <10% probability of dying within 1 year being reclassified as >10% probability. In addition, 345 (29%) of the 1206 patients initially classified as having a ≥10% probability of dying within 1 year were reclassified as having <10% probability (Table 2) [55]. Furthermore the measurement of new markers carries cost implications [41].

**Table 2:** Reclassification of patients into prognostic groups by adding two biomarkers (brain natriuretic peptide and serum troponin T) to a prognostic model for patients with heart failure [55].

Model 1 (baseline assessments)	Model 2 (baseline assessments+biomarkers)		
	Predicted probability <10%	Predicted probability ≥10%	Total
Predicted probability <10%:			
No (%) of subjects	2003 (85)	342 (15)	2345
Observed dead (%)	4.4	12.3	5.6
Predicted dead, model 1 (%)	5.7	7.8	6.0
Predicted probability ≥10%:			
No (%) of subjects	345 (29)	861 (71)	1206
Observed dead (%)	7.2	20.3	16.6
Predicted dead, model 1 (%)	13.0	16.9	15.8
Total:			
No (%) of subjects	2348 (66)	1203 (34)	3551
Observed dead (%)	4.9	18.0	9.3
Predicted dead, model 2 (%)	5.0	17.8	—

A particular motivation to update a prognostic model is to replace existing predictors that suffer from substantial inter-observer variability (such as physical examination, imaging, and histopathological techniques) [56] with more reliably measured markers. Moreover, prognostic models that include factors or markers with a causal effect on the outcome under study may be expected to be more generalisable to other populations. Such models may also be better used, since they are linked to biological (or other) pathways rather than merely based on statistical association [8]. While these suggestions are plausible, empirical evidence is lacking.

## Clinical Use of Prognostic Models

The clinical use of prognostic models should be dependent on evidence of successful validation and, preferably, on evidence of clinical impact when using the model. Not all of the models mentioned above followed this path. For example, the predictors and their weights included in the Manchester Triage System [21] were developed by medical experts without statistical modelling of patient data. It was motivated by emergency department crowding, and the aim was to shorten waiting times for those presenting with high urgency. Shortly after its development, the Manchester Triage System was introduced in various emergency departments. The Manchester Triage System is currently implemented throughout Europe. Still only limited validation studies have been performed and no impact studies. Early evaluations have focused on inter-observer agreement [57] rather than on a validation of its predictive performance [58].

One key factor for successful implementation of a prognostic model seems to be whether a model is supported by leading professionals in the field of application. For example, many prognostic models have been promoted for outcome prediction in prostate cancer with direct involvement of leading clinical investigators [18],[59]. Also, prognostic factors generally need to be readily available in routine care to allow for application of the prognostic model. That necessity may form a barrier to the use of relatively expensive or hard to access tests or new markers for prediction in primary care.

Other factors that might be associated with use of prognostic models in practice include the complexity of the model (a few or many prognostic factors) [29], the format of the model (as a score chart on paper, web based, or as standard part of an electronic patient record), the use of cut-off values for model predictions to guide decision making (rather than only providing the predicted probability), the ease of use in the consulting room, the clinical context, and the fear of “cookbook medicine” or medicolegal consequences of undue reliance on model based predictions and decisions [9],[33].

## Recommendations for Improving Prognostic Models Research

The number of published prognostic models is increasing. Unfortunately, they are often developed from poor data, inappropriately analysed, and poorly reported. For example, 10 years ago a review of 83 prognostic models in stroke found that most showed high risk of bias and serious deficiencies in statistical methods, with only four studies meeting eight simple quality criteria [14]. None had been adequately validated. A recent review of 137 studies of 101 clinical prediction rules in children, most published after 2001, showed similar methodological problems [60]. Only eight of the rules for health conditions of childhood had undergone prospective validation in broad or multiple settings. There were no impact studies. Other reviews of prognosis models across many medical areas have documented similar shortcomings [15],[61]–[63].

Clearly standards must be raised. Many of the recommendations across the PROGRESS series are relevant (see supplementary table of PROGRESS recommendations, Table S1). Here we highlight those recommendations particularly important for prognostic models.

### Clinical Impact Studies

To be useful for clinicians, a prognostic model needs to provide validated and accurate predictions and to improve patient outcomes and cost-effectiveness of care. There should be more research into understanding the impact (clinical effectiveness and costs) of using prognostic models in real world clinical practice. Clinical practice guideline recommendations relating to the use of prognostic models should be based on such impact studies (recommendation 19 in Table S1).

### Clinical Use of Prognostic Models

Easily used prognostic models may be more likely to be incorporated into clinical practice—examples include the Nottingham Prognostic Index [6], Framingham Risk Score [64], and CHADS score [65]. Indeed, some easily used models have been recommended for use without adequate evaluation. There should be more research into why some models are prematurely translated into clinical practice without adequate evaluation, whereas other models with evidence of cost effectiveness are not translated (recommendation 18).

### Statistical Methods and Data Quality in Model Development

Successful validation and clinical value are more likely when a model is developed using sound statistical methods and adequate data [3],[5],[27]. Published models have often been developed using inferior statistical methods [8],[15],[46],[61],[66]. To enhance the reliability of future models, studies should be sufficiently large and based on a study protocol including a statistical analysis plan,including careful attention to the handling of missing data and continuous predictors (recommendation 13) [5],[27],[67]. Data quality is a key aspect of developing a reliable model. Since clinically collected data may contribute many or all of the variables in a prognostic model, there should be a better understanding of the influence of clinical measurement techniques and missing observations on model performance (recommendation 20).

### Validation of Prognostic Models

It is seldom (if ever) acceptable to publish the development of a prognostic model without at least internal validation (such as cross validation or bootstrapping). Claiming that a model is clinically valuable is acceptable only with an external validation study using independent data from a different location than the development data (recommendation 9). Investigators should more often evaluate the performance of a newly developed model in a different physical location or clinical setting (recommendation 9).

### Collaboration between Research Groups

The collation and synthesis of individual participant data from multiple studies offers a natural opportunity to increase sample size [68]. Models can then be developed using data from a subset of studies and assessed on data from the remaining studies. Variation in model accuracy across studies and its causes can be explored. Also, such collaborative efforts encourage consensus towards a single well developed and validated prognostic model, rather than a number of competing and non-validated models for the same clinical problem championed by each group separately. Finally, such unification may enhance the uptake of prognostic models in practice. For example, the IMPACT consortium developed a prognostic model for mortality and unfavourable outcome in traumatic brain injury by sharing individual participant data from 11 studies (8509 patients), with successful external validation using individual participant data from another large study (6681 patients, Table 1) [43]. We encourage researchers to support collaborative efforts on data sharing to provide individual patient data to enhance the development and validation of prognostic models (recommendation 17).

### Updating a Prognostic Model

The performance of prognostic models may wane over time (for example, because diagnosis or treatments change). Also, new markers may become available. Rather than always developing new models from scratch, more often researchers should build on existing work and consider whether existing models can be improved by recalibration or adding new variables such as novel biomarkers (recommendation 21) [5],[9],[46]–[48],[69].

### Quality of Reporting

Reviews have shown widespread deficiencies in publications describing the development and validation of prognostic models [15],[61]–[63],[70]. For example, many reports fail to indicate adequately the performance of the model [62] and do not present the results in a way that can be used by clinicians [15]. Better reporting of development and validation studies is needed to help clinicians and other decision makers identify robust models with potential clinical value (recommendation 15). Consensus guidelines should be developed for reporting prognostic model research (recommendation 15), and that process is under way.

## Conclusion

Prognostic model research has three main phases: model development (including internal validation), external validation, and investigations of impact on decision making and patient outcomes [4],[8]–[10],[33]. Many prognostic models are used without clear evidence of their impact, while other well developed and validated models are not used at all. We encourage researchers to support collaborative efforts to share individual patient data allowing for both better model development and external validation. Rather than developing a steady stream of new prognostic models, researchers should shift to validation, updating, and impact studies of existing models. In the present era of biomarkers and “omics,” we encourage assessment of the extent to which new markers add value to existing models.

## Supporting Information

Table S1 **Recommendations of PROGRESS (PROGnosis RESearch Strategy).**
(DOC)

Text S1 **Review of articles in general medical journals, 2006–09.**
(DOC)
