# Supplementary material for: Prognosis Research Strategy (PROGRESS) 3: Prognostic Model Research
Source: PLoS Med. 2013 Feb 5;10(2):e1001381. doi: 10.1371/journal.pmed.1001381 (PMC3564751; doi:10.1371/journal.pmed.1001381)
Supplement: Table S1 — Recommendations of PROGRESS (PROGnosis RESearch Strategy). (DOC) [file pmed.1001381.s001.doc]

| Recommendation  (PROGRESS paper(s) introducing it) | Challenge or opportunity | Recommendation |
| --- | --- | --- |
| 1 Fundamental shift  (**1**,2,3,4) | Improvements in electronic health records, clinical imaging, and -omic technologies (genotyping and phenotyping) are beginning to challenge current disease taxonomy, clinical pre-occupation with diagnosis (rather than risk) and the focus of health care policy on process (rather than clinical outcomes). | There should be a fundamental shift in clinical practice, translational research and health care policy based on evidence from prognosis research i.e. the prospective relationships between the phenotypic, genomic and environmental assessment of people with a given startpoint and subsequent endpoints. |
| 2 Systems  (**1**,2,3,4) | Over the lifecourse individuals develop multiple diseases (both distinct and related) that often are not reflected in the current organisation of medical research or practice. | There should be an expansion of prognosis research which bridges multiple clinical specialities, health systems, pathological mechanisms, and biological systems and puts the whole patient across their ‘journey’ as the central unit of concern. |
| 3 Electronic health records  (**1**,2,3,4) | The scope and impact of prognosis research and electronic health records research (in primary and secondary care, and in disease and procedure registries) are intimately related. | There should be new programmes of methodological and empirical prognosis research exploiting electronic health records to define, phenotype and follow up people with different health related conditions. |
| 4 Field  (**1**,2,3,4) | Prognosis research is currently fragmented and not visible as a distinct entity. | Prognosis research should be recognised as a field of enquiry important in translational research, and intrinsic to the practice of clinical medicine and development of health care policy. Efforts should be made to establish prognosis research as a distinct branch of knowledge, with a set of scientific methods aimed at understanding and improving health. |
| 5 Comparing prognosis  (**1**) | The relative impact of having, compared to not having, a health condition on survival or symptom status helps identify priorities for translational research but is uncommonly reported outside the field of cancer. | There should be greater efforts to compare prognosis between those with and without a given condition, and between different conditions. |
| 6 Evidence collation  (**1**,2,3,4) | Difficulties in identifying and accessing information about prognosis, and evidence from prognosis research studies, hamper efforts to inform patients and evaluate the impact of translational efforts to improve outcomes. | Evidence from prognosis research, and information about prognosis, should be systematically collated, made easily accessible, and updated. |
| 7 Training and education  (**1**,2,3,4) | Training in how to generate or use evidence from prognosis research is currently lacking at undergraduate and postgraduate levels. | All healthcare professionals should be trained in the use of prognosis research evidence; there should be an expansion of training and education opportunities for those interested in methodological aspects of prognosis research. |
| 8 Public and patient involvement  (**1**,2,3,4) | Questions of prognosis are among the most important to patients, but the level of patient and public involvement in prognosis research is low. | Patients and the wider public should be more engaged in the goals and value of prognosis research, appropriate use of their clinical data, and better integration of patient reported outcome measures. |
| 9 Replication (validation)  (**2**,3,4) | Single studies (i.e. without replication) are commonly published on a prognostic factor, a prognostic model or a predictor of differential treatment response. Such practice is not accepted in other fields, such as genome wide association studies. | There should be greater recognition of the need for early replication studies; multiple replication at initial publication should become the standard in prognostic factor, prognostic model and differential treatment response studies. |
| 10 Quality of primary studies  (1,**2**,3,4) | Poor quality of primary studies has limited the conduct, design and interpretation of systematic reviews of prognosis research. | Initiatives to improve the quality of prognosis research through integrated standards of design, analysis and reporting should be developed across early and late stages of translation. Such standards should, where appropriate, reflect achievements in the field of randomised controlled trials, such as: protocol supported research, study registration, prospective data collection, appropriate statistical analysis, explicit and transparent reporting, and data sharing. |
| 11 Registration  (1,**2**,3,4) | Publication bias is common in prognosis research. | Registration of prognosis research in a publically accessible register (such as clinicaltrials.gov) should become more widespread. |
| 12 Protocol  (1,**2**,3,4) | Many prognosis research studies do not have a research protocol; few refer to a publicly accessible protocol. | Prognosis research studies should include a well-documented study protocol which details design and data collection methods and includes an initial statistical analysis plan. |
| 13. Statistical methods  (1,2,**3**,4) | Statistical analyses are too often deficient in prognosis research; including multiple sources of ‘significance chasing bias’, lack of appreciation of type II errors arising from small sample sizes, and the arbitrary dichotomisation or categorisation of continuous variables. | Standards in statistical analysis of prognosis research should be developed which address the multiple current limitations. In particular, continuous variables should be analysed on their continuous scale and non-linear relationships evaluated as appropriate. |
| 14 Clinical cohorts  (1,**2**,3,4) | For many diseases and health conditions there is a lack of clinical cohorts in which consented individuals are phenotyped, a biorepository established (if appropriate) and followed up for a range of health outcomes. | A comprehensive set of clinical cohorts recruiting people with specified health related condition(s) (including diagnosed disease, and symptoms) should be established as platforms for addressing a wide range of prognosis research questions. |
| 15 Reporting  (1,**2**,3,4) | Prognosis research is often poorly reported, with key information missing, or selectively included. Reporting guidelines have been developed for some types of prognosis research, but are not always implemented. | Reporting guidelines for each type of prognosis research study should be developed and implemented in order to improve transparency; identify good-quality from low-quality research; and facilitate systematic reviews, meta-analyses, and ultimately clinical decision-making. |
| 16 Language and nomenclature  (1,**2**,3,4) | Non-standard terminology hampers the field of prognosis research. | Standard terms and nomenclature should be developed and agreed in order for different clinical, translational and health care research disciplines to interact. |
| 17 Data sharing and evidence synthesis  (1,**2**,3,4) | Greater collaboration between studies and better use of existing data is important for example to achieve adequate sample sizes, provide studies for replication, and to enable more reliable evidence synthesis of prognosis studies than is currently achievable using published aggregate data. | There should be an expansion of data sharing initiatives, which include prospective individual participant data (IPD) meta-analysis, in prognosis research. |
| 18 Translational impact  (1,**2**,3,4) | There is a lack understanding of how prognosis research does, or does not lead to translational benefits at early and late stages on the pathways toward improving clinical outcome. | There should be more research into understanding what impedes, and what accelerates, appropriate translation of evidence from prognosis research at early translational stages (including discovering new intervention targets, developing new interventions, or changing the role of existing interventions) and later translational stages (such as the use of prognostic models to inform clinical decisions). |
| 19 Clinical impact studies  (**2**,3,4) | There is a lack of research evaluating the impact prognosis research on clinical decision making, health care policy, and on clinical outcomes. | There should be more research quantifying the impact (clinical effectiveness and costs) of implementing the findings of outcomes research, prognostic factors, prognostic models, and approaches to stratified medicine in real world clinical practice. |
| 20 Data quality  (1,2,**3**,4) | Clinically collected data is central to prognosis research, and the implementation of prognosis research findings, but the quality of such data needs to improve. | There should be greater efforts to understand and improve the quality of clinically collected data, including standardising methods of measurement and prevention of missing values. |
| 21 Updating  (**3**,4) | Changes in clinical care, the absolute risks of endpoints and the ability to measure new potential prognostic factors pose a challenge of updating in prognosis research. Too often new prognostic models are developed rather than updating existing ones. | There should be a greater recognition of the need for updating of prognostic models and other forms of prognosis research. |
| 22 Stratified medicine: research designs  (3,**4**) | Research and analyses to identify factors that predict treatment response are often flawed, as they only assess either (i) patients receiving a treatment, or (ii) patients with positive factor values. | Robust randomised trials to identify factors that truly predict differential treatment response should be encouraged. In the case of a truly binary predictor, such trials involve in four groups of randomised patients: some patients with negative factor values in the control group, some with negative values in the treatment group, some with positive values in the control group and some with positive values in the treatment group. |
| 23 Stratified medicine: impact  (3,**4**) | Bold claims are made for the emerging ability to target interventions at sub-groups of patients (‘stratified medicine’) based on biologically relevant predictors of differential treatment response. | There should be rigorous evaluation of ‘personalised medicine’ approaches on health outcomes, including comparison of approaches based targeting intervention (with prognostic models or factors that predict differential treatment response) and ‘all comer’ approaches. |
| 24 Industry  (1,2,3,**4**) | Industry (drug, device, biomarker, IT) interest in prognosis research including tests for stratified medicine (sometimes called ‘companion diagnostics’), drug safety, outcomes research, and real world evidence is growing. | Appropriate models of industry and publicly funded prognosis research should be developed which allow unbiased inference. |
