# Supplementary material for: Prognosis Research Strategy (PROGRESS) 3: Prognostic Model Research
Source: PLoS Med. 2013 Feb 5;10(2):e1001381. doi: 10.1371/journal.pmed.1001381 (PMC3564751; doi:10.1371/journal.pmed.1001381)
Supplement: Text S1 — Review of articles in general medical journals, 2006–09. (DOC) [file pmed.1001381.s002.doc]

We updated a previous review by Reilly and Evans[33] in four general medical journals (*NEJM*, *JAMA*, *BMJ*, *Annals of Internal Medicine*), which in turn extended the reviews by Wasson[71] for the years 1981-84 and by Laupacis[72] for the years 1991-1994 (both in the same four journals). Although we acknowledge that these general journals do not encompass the majority of prognostic models, we repeated the search for the years 2006-2009 in the same general medical journals and also included *Lancet* and *PLoS Medicine*. We used a previously published search strategy for prognostic models.[73]

For this paper, we identified publications describing the development, external validation, or impact assessment (or combination) of a prognostic model. No other selection criteria were used. External validation studies had to include patients that were not included in the statistical development of the model (e.g. selected from another practice or hospital). However, temporal validation (including splitting a large data set in an older development part and more recent validation part) was considered as external validation.[9] [32] Assessment of the model’s impact considered the model’s ability to change clinical decision making and/or patient outcome. Impact studies could take the form of a modelling study (e.g. using a Markov or other type of decision analytic model), or be based on empirical data, such as a (cluster) randomised or before-after study.[9]

The search resulted in 1257 hits in the six journals for the years 2006-2009, and identified 71 original publications reporting on a prognostic model, either on a model development, external validation, impact assessment or a combination of these. The numbers of studies for each type are shown in Figure 3, along with the findings of the three previous reviews. Of the 61 publications in the new cohort reporting model development studies 13 also reported an external validation, leaving 8 (21−13) pure external validation studies.
